# Tendon Reconstruction With Cadaveric Allograft in a Patient With Flexor Tendon Injury Without Treatment for Six Months

**DOI:** 10.7759/cureus.18004

**Published:** 2021-09-15

**Authors:** Carolina Lizarzaburu-Ortiz, Daniela Dominguez, Ian Valdez, Eduardo Rojas, Diego J Lizarzaburu

**Affiliations:** 1 Plastic and Reconstructive Surgery, Military Hospital, Quito, ECU; 2 Medicine, Universidad Internacional del Ecuador, Quito, ECU; 3 “Incubadora de Investigación en Medicina” (InMed), NeurALL Nest, Quito, ECU; 4 Plastic and Reconstructive Surgery, Universidad Católica del Ecuador, Quito, ECU

**Keywords:** allograft, tendon injury, case report, cadaveric graft, flexor tendons

## Abstract

We present the case of an 18-year-old male patient with a penetrating lesion at zone II of the flexor compartment of the left hand. During surgery, complete reabsorption of the second deep and superficial flexor tendons was evidenced, prompting the decision to perform a two-stage procedure. First, a spacer was placed, and pulley reconstruction was performed. Six weeks later, we placed a cadaveric allograft and splint with the Kleinert-Duran technique for proper rehabilitation. Our case report highlights that a two-stage procedure with an allograft is a feasible technique resulting in good post-surgical functional status despite tendon reabsorption and six months between trauma and surgical intervention.

## Introduction

Flexor tendon injuries significantly impact a patient’s quality of life, resulting in significant functional deficits. Its specific prevalence has not been reported [1,2]; however, in general, acute tendon injuries have an incidence of 33.2 per 100,000 person-years as reported in a Midwest county in the United States from 2001 to 2010 [3].

Tendinous lesions in Verdan’s zone II are challenging as adhesions of the repaired tendon within the synovial sheath can occur [4]. However, the placement of tendon grafts for the reconstruction of lesions within this area has shown good results [5]. Delayed secondary tenorrhaphy (DST) in the setting of flexor tendon reabsorption due to a penetrating trauma is a real surgical challenge [6]. Over the years, significant development has occurred regarding tendon suture techniques, incisions, rehabilitation methods as well as the availability of a myriad of choices in terms of materials or tendon grafts for repair [5]. Tendon grafts can come in various forms, such as autografts, synthetic grafts, living donor allografts, and even cadaveric allografts [7]. For the latter, there are records dating back to 1967 where the first attempts to repair a flexor tendon with a cadaveric allograft were performed by Peacock [8,9]. To date, records of such a procedure are scarce, which makes the use of a cadaveric tendon allograft an area worth exploring, particularly in patients that present with tendon reabsorption.

The patient was informed that data concerning the case would be submitted for publication, and he provided consent.

## Case presentation

**Figure 1:**
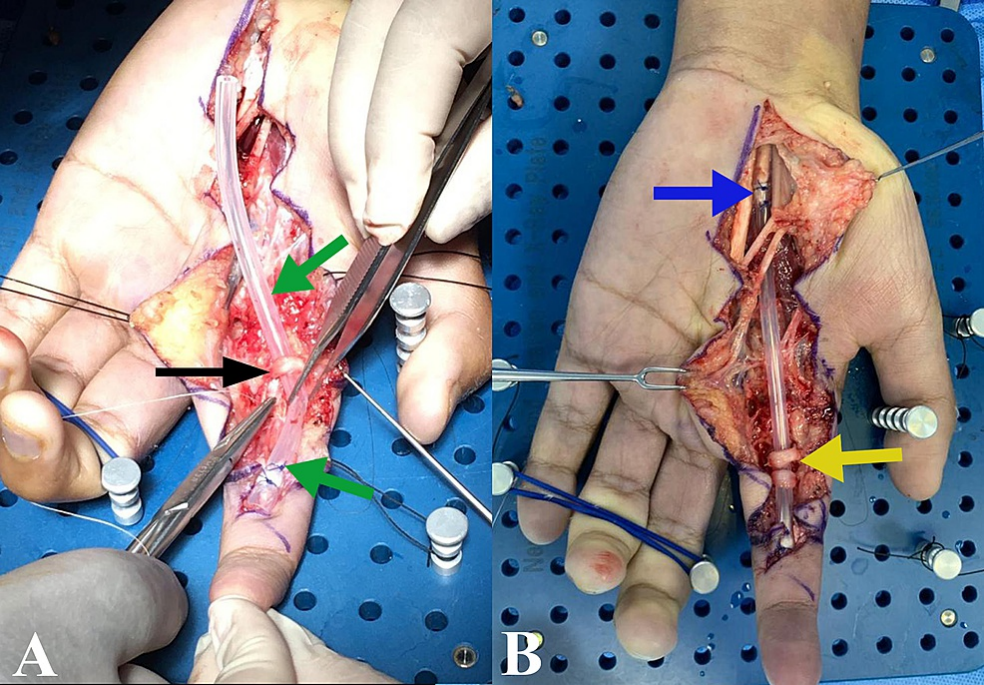
Silicone spacer placement (A) Silicone spacer placement from intermediate phalanx (green arrow) and A2 pulley rebuild (black arrow). (B) Brunner type incisions, A1 pulley rebuild (yellow arrow), and silicone spacer placement proximal to the transverse carpal ligament (blue arrow).

We present an 18-year-old male patient, who six months before consulting our service, suffered a penetrating trauma injuring the flexor digitorium superficialis and flexor digitorium profundus tendons of the index finger of the left hand. After this injury, the patient reported receiving no medical care for it and had a negative personal or family history of collagen diseases. Physical examination revealed movement limitation during flexion of the index finger of the left hand, and a small scar measuring 5cm, located in Verdan`s flexor zone II-III (near the distal palmar crease). In the first intervention, Brunner-type incisions were made extending from Verdan’s flexor zone II to IV to widen the surgical field. We verified that tendons were replaced with a scar and fibrotic tissue. It is assumed that, due to the elapsed time from trauma, tissue was reabsorbed due to lack of movement and destruction of the tendon sheath, which compromised tendon nutrition. This was further evidenced during surgery where we found reabsorption of the pulleys and absence of the tendons, from the middle phalanx until the transverse carpal ligament (flexor retinaculum). It should be noted that, during surgery, we confirmed that tendons were not retracted into the forearm, but rather were partially reabsorbed. Additionally, a tubular silicone spacer (fashioned from the flexible tubing of a Jackson-Pratt drain, BRANDEN® measuring 127mm) was placed, joining the distal and proximal ends of the resorbed tendon in order to enable the formation of a synovial pseudo-sheath. Ring pulleys A1 and A2, from the index finger, were reconstructed by using fibrotic tendon sheath segments (Figures 1A, 1B).

**Figure 2:**
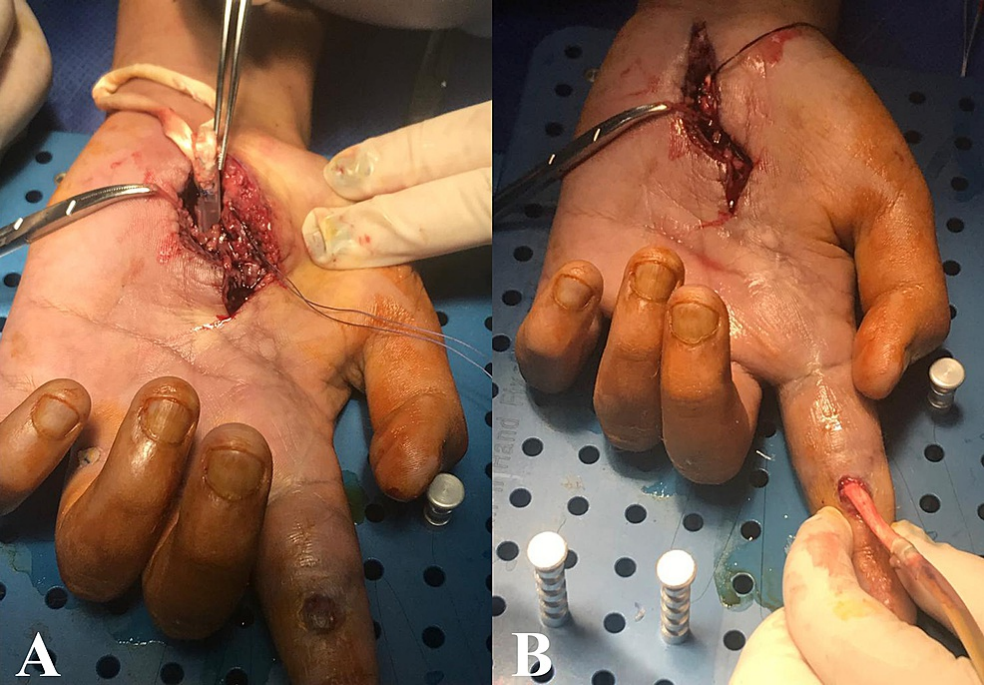
Cadaveric allograft placement (A) Replacement of the silicone spacer by cadaveric tendon graft. (B) Cadaveric tendon graft coming out at the level of the intermediate phalanx.

In the immediate postoperative period, a Kleinert-Duran splint was used to promote early rehabilitation with passive movement of the digital joints for six weeks, in order to prepare for the second intervention, achieving at the end full passive range of motion (ROM). Three months later, the second intervention was performed with two incisions in Verdan`s flexor zone II and IV of the left hand, replacing the tendon silicon spacer with a cadaveric allograft (Figure 2A). The cadaver allograft used came from the Achilles tendon and was obtained from a tissue bank that assures the quality and sterilization of its biological material by using universal standards of management as described in the discussion section. Tenorrhaphy with 4-0 Prolene was performed at the proximal remains of flexor digitorum profundus in zone IV with the cadaveric tendon graft (Figure 2A), and the spacer was removed by the distal end, dragging the cadaveric graft through the canal formed in the first surgical intervention. The distal end of the graft was separated from the spacer and sutured to the intermediate phalanx (Figure 2B). The flexor tendon graft was tensioned with the rebuilt pulleys A1 and A2.

**Figure 3:**
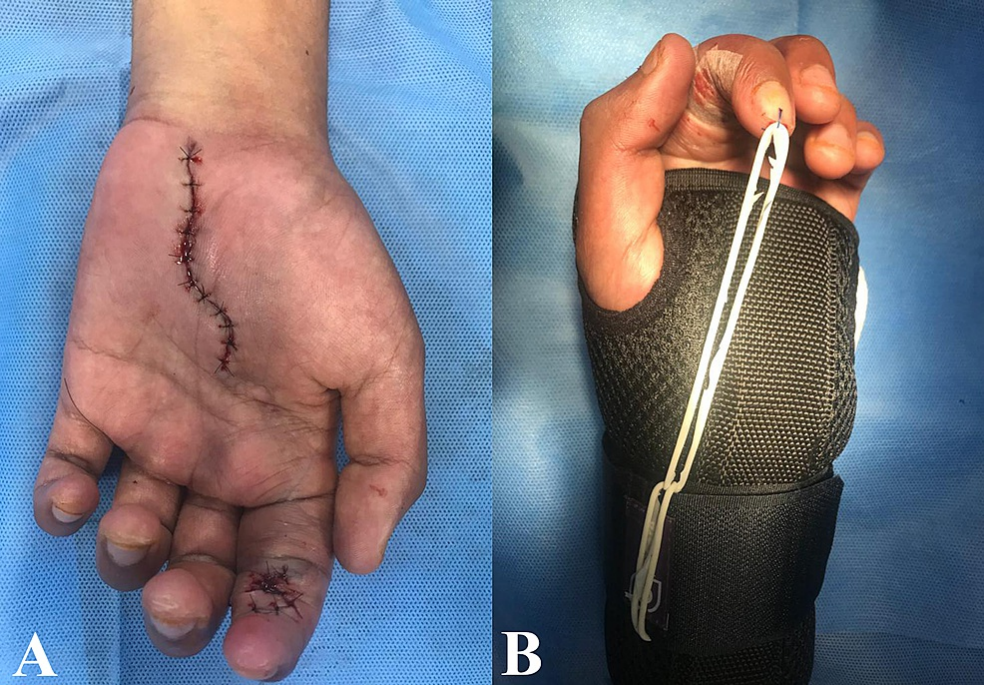
Final postoperative results (A) Postsurgical sutured wounds. (B) Kleinert-Duran splint.

Finally, in the immediate postoperative period, the Kleinert-Duran splint was placed (Figures 3A, 3B), with verification of flexion movement of the affected finger. Post-op rehabilitation consisted of a dorsal block with a splint for three weeks, limited active and passive extension with traction through elastic bands extending from the nail to the wrist. Then, by six to eight weeks, gentle and active bending exercises with gradually increasing resistance were performed. Once the healing of the skin was completed, active rehabilitation began; obtaining appropriate arch flexion, according to Strickland criteria [10] and active ROM was almost perfect with good postoperative results.

No immunosuppressive pharmacological treatment was prescribed. The patient’s functional recovery was remarkable. Video 1 showcases the patient’s functional recovery six months after surgery. One year later, he can perform cascading movements of the fingers with slightly greater flexion than normal. Additionally, prehension of the hand was recovered resulting in minimal postoperative morbidity.

**Video 1:** Six-month functional recovery

## Discussion

The goal of any flexor tendon reconstruction procedure is to restore its original function. However, there are several challenges along the way. Our first drawback was the absence of surgical intervention six months after the injury. In the literature, the optimal time for surgical intervention is stated as being two weeks after injury, because from the third-week problems such as localized inflammation, absorption, adherence of the tendon, fibrosis of the tendon sheath, a greater probability of infection, and tendon rupture increase significantly [11]. To reconstruct the flexor tendons in two stages, a fragment of the deep tendon is preserved for distal suturing to a silicone rod, which creates an empty space that will be occupied by the graft during the second surgical time [6]; also, the pulleys that are important fixation systems are reconstructed. Finally, tendon grafts or bench flexor tendons can be used for the final repair [12]. As stated before, the placement of the silicon spacer allows the creation of a synovial pseudo-sheath around it, with intima, media, and adventitia layers. These provide vascular nutrition, functional and structural support for the tendon graft to be placed afterward [13]. This synovial pseudo-sheath provides multiple benefits such as allowing for proper gliding of the tendon and minimization of the risk of adhesions [13]. However, an alternative is the reconstruction of the synovial sheath by using flaps of various origins, such as the dorsal podium, the fascia lata, or the temporal fascia; however, the drawbacks of this technique include morbidity at the donor site, higher likelihood of the patient not consenting to such procedure, and low availability of donor sites [13].

In our case, the choice of a cadaveric allograft was clear due to the benefits described above regarding the two-stage technique and because the waiting list was not significant. The tendon of cadaveric origin has been shown to retain an astonishing level of strength, compared to the tendon of a living patient [14]. Important issues to consider are the risk of graft rejection and infection transmission. Regarding graft rejection, it should be noted that different tissues have a different number of cells, with different mechanisms of antigen presentation and expression [15]. Fortunately, tendons are mostly structural tissues, with low cellularity which enables tendon grafts to have a low probability of being rejected due to their relatively low antigenicity [15]. However, the nutritional demand is high and must be satisfied by the tendon pseudo-sheath constructed thanks to the spacer placed during the first surgical intervention [5,7]. On the other hand, infections with the highest risk of transmission include HIV and Hepatitis C [16]. For this reason, proper safety control in the tissue bank and rigorous protocols to minimize the risk of infection should be in place. These begin with the extraction of the tendon graft using a sterile technique, followed by washing, placement of the tendon in nutritional substances, sealing in sterile bags, and freezing the specimen at temperatures of -80 degrees [17]. When the graft is going to be used, 30 minutes before, it is washed with saline solution and placed in an antibiotic solution prior to placement in the patient [17]. In addition, different mechanisms of colonization of the cells from the recipient to the graft have been theorized, thereby further reducing the risk of graft rejection [16,17]. An alternative for preventing rejection includes the decellularization of the graft, which has been proven to not affect tendon strength at all [18].

In terms of patient rehabilitation, at the end of the second surgical stage, physical therapy began with the Kleinert-Duran splint and then with active movements. The addition of active flexion to the modified Kleinert regime significantly improves the recovery of the original function of the digital flexor tendon, increases grip strength, and accelerates recovery with better results when compared to the modified Kleinert regime [19]. Early mobilization is critical as it prevents the formation of adhesions and joint stiffness [20].

## Conclusions

In the presented two-stage surgical case, a sterile silicone tendon spacer was used, pulleys A1 and A2 were reconstructed, and a cadaveric tendon graft was placed with a favorable evolution of flexor mobility of the second finger of the left hand. The advantages of this delayed secondary flexor tendon reconstruction with cadaveric allograft procedure include the reduction of surgical times, higher number of grafts available for reconstruction with varying lengths, decreased sequelae at the donor site, and complex reconstructions can be performed relatively easy with minimal morbidity if implementing proper physical therapy and an early mobilization protocol postsurgically.

## References

[1] Chang MK, Tay SC (2018). Flexor tendon injuries and repairs: a single centre experience. J Hand Surg Asian Pac Vol.

[2] Manninen M, Karjalainen T, Määttä J, Flinkkilä T (2017). Epidemiology of flexor tendon injuries of the hand in a northern Finnish population. Scand J Surg.

[3] de Jong JP, Nguyen JT, Sonnema AJ, Nguyen EC, Amadio PC, Moran SL (2014). The incidence of acute traumatic tendon injuries in the hand and wrist: a 10-year population-based study. Clin Orthop Surg.

[4] Beredjiklian PK (2003). Biologic aspects of flexor tendon laceration and repair. J Bone Joint Surg Am.

[5] Chattopadhyay A, McGoldrick R, Umansky E, Chang J (2015). Principles of tendon reconstruction following complex trauma of the upper limb. Semin Plast Surg.

[6] Battiston B, Triolo PF, Bernardi A, Artiaco S, Tos P (2013). Secondary repair of flexor tendon injuries. Injury.

[7] Samora JB, Klinefelter RD (2016). Flexor Tendon Reconstruction. J Am Acad Orthop Surg.

[8] Peacock EE Jr, Madden JW (1967). Human composite flexor tendon allografts. Ann Surg.

[9] Tobin GR, Breidenbach WC 3rd, Ildstad ST, Marvin MM, Buell JF, Ravindra KV (2009). The history of human composite tissue allotransplantation. Transplant Proc.

[10] Tang JB (2005). Clinical outcomes associated with flexor tendon repair. Hand Clin.

[11] Sade I, İnanir M, Şen S, Çakmak E, Kablanoğlu S, Selçuk B, Dursun N (2016). Rehabilitation outcomes in patients with early and two-stage reconstruction of flexor tendon injuries. J Phys Ther Sci.

[12] Azari KK, Meals RA (2005). Flexor tenolysis. Hand Clin.

[13] Al-Qattan MM, Al Mohrij SA (2019). A modified technique of two-staged extensor tendon reconstruction in zones 6-8 in a patient with absent palmaris/plantaris tendons: a case report. Int J Surg Case Rep.

[14] Yang M, Wang Z, Li Y, Guo B (2013). Bilateral cadaveric Achilles tendon graft in reconstruction after Achilles tendon tumor resection. J Foot Ankle Surg.

[15] Llull R (1998). An open proposal for clinical composite tissue allotransplantation. Transplantation Proc.

[16] Asencio G, Abihaidar G, Leonardi C (1996). Human composite flexor tendon allografts. A report of two cases. J Hand Surg Am.

[17] Xie RG, Tang JB (2012). Allograft tendon for second-stage tendon reconstruction. Hand Clin.

[18] Fox PM, Farnebo S, Lindsey DP, Chang J, Bosque T, Chang J (2013). Decellularized human tendon-bone grafts for composite flexor tendon reconstruction. J Hand Surg Am.

[19] Rigó IZ, Haugstvedt JR, Røkkum M (2017). The effect of adding active flexion to modified Kleinert regime on outcomes for zone 1 to 3 flexor tendon repairs. A prospective randomized trial. J Hand Surg Eur Vol.

[20] Chinchalkar SJ, Larocerie-Salgado J, Suh N (2016). Pathomechanics and management of secondary complications associated with tendon adhesions following flexor tendon repair in zone II. J Hand Microsurg.

